# Long-Term Capsaicin Administration Ameliorates the Dysfunction and Astrogliosis of the Brain in Aged Mice with Missing Maxillary Molars

**DOI:** 10.3390/nu15112471

**Published:** 2023-05-25

**Authors:** Masae Furukawa, Hirobumi Tada, Resmi Raju, Jingshu Wang, Haruna Yokoi, Yoriko Ikuyo, Mitsuyoshi Yamada, Yosuke Shikama, Kenji Matsushita

**Affiliations:** 1 Department of Oral Disease Research, Geroscience Research Center, National Center for Geriatrics and Gerontology, Obu 474-8511, Japan; 2 Department of Nutrition, Faculty of Wellness, Shigakkan University, Obu 474-8651, Japan; 3 Department of Integrative Physiology, Geroscience Research Center, National Center for Geriatrics and Gerontology, Obu 474-8511, Japan; 4 Department of Operative Dentistry, School of Dentistry, Aichi Gakuin University, Nagoya 464-8651, Japan

**Keywords:** capsaicin, GFAP, s100β, TRPV1, mastication, brain aging

## Abstract

Tooth loss and decreased masticatory function reportedly affect cognitive function; tooth loss allegedly induces astrogliosis and aging of astrocytes in the hippocampus and hypothalamus, which is a response specific to the central nervous system owing to homeostasis in different brain regions. Capsaicin, a component of red peppers, has positive effects on brain disorders in mice. Decreased expression of transient receptor potential vanilloid 1, a receptor of capsaicin, is associated with the development of dementia. In this study, we investigated the effect of capsaicin administration in aged mice (C57BL/6N mice) with reduced masticatory function owing to the extraction of maxillary molars to investigate preventive/therapeutic methods for cognitive decline attributed to age-related masticatory function loss. The results demonstrated that mice with impaired masticatory function showed decreased motor and cognitive function at the behavioral level. At the genetic level, neuroinflammation, microglial activity, and astrogliosis, such as increased glial fibrillary acidic protein levels, were observed in the mouse brain. The mice with extracted molars fed on a diet containing capsaicin for 3 months demonstrated improved behavioral levels and astrogliosis, which suggest that capsaicin is useful in maintaining brain function in cases of poor oral function and prosthetic difficulties.

## 1. Introduction

Since the life expectancy of most individuals has increased to 100 years, preserving quality of life is important [1]. Eating well is important to sustain a good quality of life; thus, retaining one’s teeth and maintaining masticatory function is important. Oral malfunction, especially tooth loss, affects not only masticatory function but also cognitive function [2,3]. Individuals with a greater amount of tooth loss have poorer cognitive function than those with lesser tooth loss; discrete data suggest that dementia progresses with a decline in oral health [4,5,6,7,8]. Prosthetic replacement of missing teeth and masticatory exercises reportedly improve cognitive function [9]. 

Prosthetic treatments are necessary for replacing missing teeth to sustain masticatory function; however, dental treatment is challenging in cases such as bedridden patients who are unable to visit the dentist [10], patients with dementia [11], and those with a heightened gag reflex. 

Dental care for patients with dementia presents several problems [12,13]. Owing to their impaired communication skills, both dental treatment and oral care could be challenging. Moreover, some patients are disturbed by minor changes in the oral environment, such as new dentures, and have difficulty adjusting to these changes [12]. Patients with severe dementia often experience oral motor deficits, making dental management and denture stabilization challenging [12,13].

The marked proliferation of astrocytes in the brain, known as astrogliosis, has been reported in patients with dementia [14]. Astrogliosis presents in various forms with varying intensities of astrogliosis, such as increased expression of glial fibrillary acidic protein (GFAP) and cell hypertrophy [14]. In addition, GFAP expression increases with aging of the astrocytes [15,16,17]. To date, we have demonstrated that tooth loss owing to maxillary molar enhances astrogliosis [3]. 

Capsaicin, which is abundantly present in chili peppers, has been found to promote the swallowing reflex in the oral cavity [18]. Capsaicin binds to the transient receptor potential vanilloid 1 (TRPV1) receptors and releases substance P from the sensory nerves of the pharynx into the mucosa. This increases substance P concentrations, which, in turn, facilitates the triggering of reflexes. Capsaicin has also been shown to play an important role in a variety of other medicinal effects, including anti-inflammatory [19], anticancer [20], analgesic [21], cardioprotective [22], antioxidant [23], anti-obesity, antidiabetic, antibacterial, antifungal, and anticoagulant function [23,24].

Previously published studies have reported the safety and use of 0.01% capsaicin [25].

More recently, capsaicin has been considered a potential therapeutic agent for Alzheimer’s disease (AD) by reducing cognitive impairment [26] and regulating microglial autophagy via TRPV1 [27].

Subtype glutamate receptor 2 (GluA2) of the α-amino-3-hydroxy-5-methyl-4-isoxazole propionic acid-type glutamate receptor (AMPA)-type glutamate receptor, which is involved in the rapid neurotransmission in the central nervous system, is related to TRPV1 [26].

In this study, we investigated preventive or therapeutic mechanisms of cognitive decline caused by age-related decline in masticatory function. In addition, we examined the effects of capsaicin administration in aged mice with masticatory dysfunction attributed to the extraction of the maxillary molars.

## 2. Materials and Methods

### 2.1. Animals

We used 18-month-old male C57BL/6N mice (*n* = 10 per group) in this study; female mice were not used in the experiments owing to their sex cycle. The animals were kept in an air-conditioned, clean room at the National Center for Geriatric Research and Gerontology (NCGG). All the experiments were approved by the Animal Welfare Committee of the NCGG (Approval No. 4-10-R1). Mice were kept under a 12 h light/dark cycle, with lights turned off from 7:00 p.m. to 7:00 a.m.

Mice were randomly divided into three groups: control (CC, control diet *n* = 10), extraction (CE, control diet *n* = 10), and capsaicin diet extraction (CAPE, capsaicin 0.01% diet group *n* = 10).

The CC and CE groups were fed standard laboratory pellets (D11112201N; Research Diets, Tokyo, Japan) and water *ad libitum*. The CAPE group was regularly fed capsaicin pellets (D21122701, capsaicin-added feed, open standard diet feed +0.01% capsaicin added, red, Research Diets). The CE and CAPE groups had their upper first molars extracted after a week of habituation. The extraction procedure for maxillary molars was in accordance with our previous literature [3]. Mice were fixed on a special fixator (NAIGAI-CFK-2S, AS ONE Corporation, Osaka, Japan) in the small animal experiments. In the extraction group, maxillary bilateral first molars were extracted by dislodging them using a dental explorer. After tooth extraction, an anesthetic antagonist was administered; no analgesia was administered. These procedures took up to 30 min. No analgesics or anti-inflammatory medications were administered postoperatively.

Subsequently, we evaluated the behavioral tests, as well as the feed and water intake, of each group during the third month of rearing. Each mouse was subjected to behavioral experiments and euthanized after 3 months.

Mice were under anesthetized (medetomidine hydrochloride 0.9 mg/kg + midazolam 12 mg/kg or alfaxalone 90 mg/kg + butorphanol 15 mg/kg diluted with saline and intraperitoneal injection), and the brains were harvested after euthanasia by cervical dislocation.

All the experiments were conducted in accordance with the Guide for the Care and Use of Laboratory Animals, published by the National Institutes of Health (NIH Publication, eighth edition, 2011). In addition, this study was conducted in accordance with the Animal Research: Reporting of In Vivo Experiments Guidelines.

### 2.2. Body Weight and General Health

After a week of habituation, all the mice were weighed using a precise laboratory scale with an accuracy of 0.01 g, and they were examined for any indications of poor health, such as wounds and deteriorated physical condition. Body weight measurement was performed on a weekly basis.

### 2.3. Blood Tests

#### 2.3.1. Preparation of Blood Serum

In this study, mice were euthanized between 10:00 a.m. and 12:00 p.m. for blood collection. They were rapidly decapitated, and trunk blood (approximately 1 mL) was collected into centrifuge tubes (CJ-2AS, Terumo, Tokyo, Japan) and centrifuged (3000× *g*, 10 min, 15 °C) for serum separation. The collected serum was divided into two purposes: (i) biochemical test and (ii) corticosterone assay. The serum samples were stored at −80 °C until the assay. 

#### 2.3.2. Serum Biochemistry Test

Serum physiological (albumin, total cholesterol [T-CHO]) tests were performed at the Nagahama Biological Science Laboratory (Nagahama, Shiga, Japan) of Oriental Yeast Co., Ltd. We analyzed at least six mice per group for this test.

### 2.4. Twenty-Four-Hour Locomotor Activity

Locomotor activity was measured as specified by Yamasaki et al. [28]. Each mouse (*n* = 8) was housed separately in a cage under a 12 h light/dark cycle and subsequently bred individually. Food and water were provided *ad libitum*. We determined the amount of exercise in the breeding cage using an infrared photobeam sensor (Time HC8 Single; O’ Hara & Co., Ltd., Tokyo, Japan) and calculated the total distance moved by the mice per hour.

### 2.5. Y-Maze Test

We used the Y-maze test to evaluate the mechanisms by which tooth loss and capsaicin rearing affect the learning and memory performance in mice. We included at least five mice per group for this test. First, we assessed spontaneous alternation behavior in a gray acrylic Y-shaped maze consisting of three arms (60 cm long × 60 cm wide × 25 cm deep, YM-3002, O’ Hara & Co., Ltd.) projecting from each side of a central equilateral triangle. The Y-maze test was performed as described by Wahl et al. [29]. The learning rate is based on the number of sessions required to meet this criterion. A mouse was placed in one arm (No. 1), where it received the following three options as its first choice: staying in arm one, moving to arm two, or moving to arm three. An alternation was considered correct if the mouse visited a different arm and did not return to the two previously visited arms. We calculated the ratio of correct alternations to the number of visits during a 10 min observation period as the frequency of alternation. A frequency >50% indicated spontaneous alternation. The test was repeated, and the number of correct responses was used as a measure of memory. Percentage alternation was the dependent variable [29], which was calculated as follows:

((the number of alternations)/(total entries − 2)) × 100.

### 2.6. Motor Skill Learning Test

A rotarod machine with automatic timers and falling sensors (MK-660D; Muromachi Kikai, Tokyo, Japan) was employed. We analyzed at least five mice per group for this test. We followed the standard operating procedure of the RIKEN BioResource Center (Saitama, Japan). Each mouse was placed in a drum with a diameter of 9 cm. The drum surface was covered with hard chloroethylene to prevent surface gripping. The unit was set to accelerate from 4 to 40 rpm in 300 s. Following the drop, the mice were allowed to rest for 20 min and subsequently returned to the drum (a maximum of twice in one session). To evaluate long-term memory, we repeated the test once daily for two consecutive days. Daily latency was recorded automatically on the rod until the mouse fell.

### 2.7. Real-Time Polymerase Chain Reaction (PCR)

For PCR analysis, a minimum of six mice per group were examined. To investigate the molecular changes in the brain following the reduction of mastication and administration of capsaicin, we assessed the gene expression related to memory and cognitive functions in the hypothalamus and hippocampus using real-time PCR. Initially, total RNA was extracted from the hypothalamus and hippocampus using the NucleoSpin RNA kit (cat. U0955C; Takara Bio Inc., Shiga, Japan) as per the manufacturer’s instructions. The total RNA concentration was adjusted to 100 ng/μL using a NanoDrop ^TM^2000 spectrophotometer (Thermo Fisher Scientific, Waltham, MA, USA). Subsequently, first-strand cDNA synthesis was carried out using the ReverTra Ace-α Kit (TOYOBO, Osaka, Japan). PCR amplification was performed utilizing the FastStart Essential DNA Green Master (Roche, Mannheim, Germany) according to the manufacturer’s protocol, with detection on a LightCycler 96 System (Roche). Table 1 summarizes the primer sequences. The expression of the target gene was normalized to that of the housekeeping gene glycer-aldehyde-3-phosphate dehydrogenase, and the results were reported upon comparison with the control for each sample. These experiments were conducted in triplicates under each condition. The values are presented as fold-changes between samples using the 2^–ΔΔCt^ method [30]. 

### 2.8. Immunohistochemistry

We conducted immunohistochemistry analysis on a minimum of three mouse brains per group and performed quantification on at least 10 sections per mouse. The brains were extracted from experimental mice and placed in phosphate-buffered saline (PBS) for 24 h. Serial coronal sections (50 μm) were obtained at the bregma level (1.5–2 mm posterior ±1.2–1.3 mm mediolateral) and processed for immunohistochemistry using VT1200S (Leica Microsystems, Wetzlar, Germany). To block nonspecific binding, the brain slices were treated with PBS containing 1% Triton X-100 and 10% normal donkey serum (S30-100 mL, Merck, Darmstadt, Germany) for 1 h. The brain sections were incubated overnight at 4 °C with specific primary antibodies diluted in PBS. The primary antibodies used were as follows: anti-GFAP (1:1000; ab4674, Abcam, Cambridge, UK), anti-s100β (1:200, ab52642, Abcam), anti-Iba-1 (1:200; ab178846, Abcam), anti-NeuN (1:1000; ab104224, Abcam), anti-TRPV1 (1:500; 66983-1-IG, PGI), and anti-GluA1 (glutamate ionotropic receptor AMPA-type unit 1) (1:100; ab31232, Abcam). Following incubation with the primary antibody, the tissue slices were washed thrice with PBS and subsequently incubated with subtype-specific fluorescent secondary antibodies, i.e., goat anti-rabbit immunoglobulin G (IgG) H&L (Alexa Fluor^®^ 488) (1:200, ab150077, Abcam) and donkey anti-mouse IgG (H + L) (Alexa Fluor 555) (1:200; ab 150110, Abcam) for 1 h at 25 °C with shaking. Nuclei were stained with 4′,6-diamidino-2-phenylindole (D523, Dojindo, Kumamoto, Japan). The controls were incubated in the absence of primary antibodies. The sections were observed under a Keyence BZ-X800 microscope (KEYENCE Co., Osaka, Japan). We calculated the number of positive cells in the hippocampal cornu ammonis region using ImageJ software v1.52a (NIH, Baltimore, MA, USA), and three individuals performed the quantitative analysis of each immunolabeled area. We randomly selected 4 fields (1 × 1 mm^2^ square per field) of the hippocampus, and the mean values were used for the statistical analysis. Measurements were obtained from 10 sections per mouse, and all the immunostaining procedures were performed in a blinded manner. Three individuals performed the quantitative analysis for each immunolabeled region.

#### DAPI Staining and Normalization

For nuclear staining and normalization, DAPI staining was performed on the same sections. DAPI-stained sections were imaged using a fluorescence microscope equipped with a DAPI filter. Quantitative analysis of immunohistochemical staining was performed using ImageJ software v1.52a (NIH, Baltimore, MA, USA). Regions of interest (ROIs) were selected within the hippocampus, and the intensity or area of the target protein staining was measured. The number of DAPI-stained nuclei within the same ROIs was simultaneously counted using image analysis software. The immunohistochemical staining values were then normalized to the number of nuclei detected by DAPI staining to account for the variations in cell density or tissue size.

### 2.9. In Vitro Assay

Murine astrocytes from the C8-D1A cell line (ATCC^®^ CRL-2541; Manassas, VA, USA) were used in in vitro experiments. The cells were maintained in Dulbecco’s Modified Eagle’s medium (DMEM) supplemented with 10% fetal bovine serum, 2 mM L-glutamine (ATCC^®^ 30-2002™, Manassas, VA, USA), 100 U/mL penicillin, and 100 μg/mL streptomycin (Wako, 168-23191, Osaka, Japan). The cells were obtained after two passages (passaged three times before the experiment) and cultured at 70% confluence in 100 mm diameter culture dishes. They were then transferred to 6-well flat-bottom culture plates for subsequent experiments. Capsaicin was purchased (Sigma-Aldrich, Inc., M2028, St. Louis, MO, USA) and dissolved in ethanol for use in a culture medium. Cells were incubated at 37 °C in a humidified chamber containing 5% CO_2_. After incubation, the culture was transferred to 96 wells for the 3-(4,5-dimethylthiazol-2-yl)-5-(3-carboxymethoxyphenyl)-2-(4-sulfophenyl)-2H-tetrazolium assay to check the 25 μM concentration of capsaicin (data not shown) [31]. For astrocyte aging, 75 μM H_2_O_2_ (Wako, 084-07441) was added and incubated for 2 h, followed by a medium change [32]. The capsaicin-supplemented group was changed to capsaicin-supplemented DMEM during the medium change. RNA was extracted after 48 h from cultured astrocytes using 25 μM capsaicin [31], and *Gfap* and *Trpv1* mRNA expression was confirmed by PCR.

### 2.10. Statistical Analyses

Data were analyzed by analysis of variance (ANOVA), followed by a post hoc test or Student’s *t*-test, using the program GraphPad Prism 5.0 (GraphPad Prism Software Inc., San Diego, CA, USA). The F-value was calculated, indicating a significant effect. Results are expressed as mean ± standard error of mean, and *p* < 0.05 was considered statistically significant.

## 3. Results

### 3.1. Changes in Body Weight Owing to Loss of Maxillary First Molars and Effect of Capsaicin Diet

**Figure 1:**
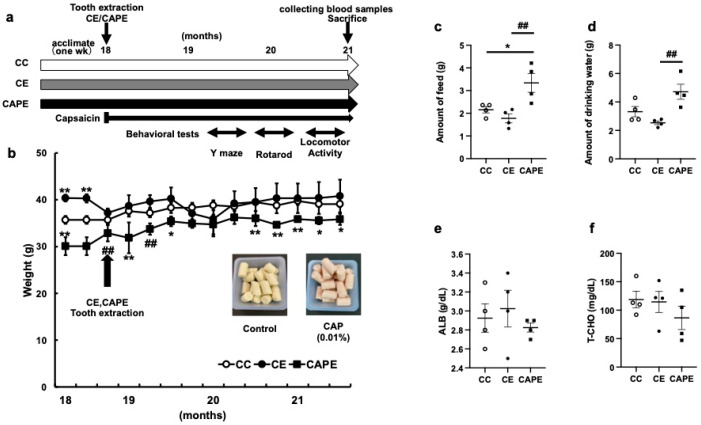
Weight and capsaicin feeding changes owing to loss of the maxillary first molars. (**a**) Study design. (**b**) Weight change: The arrows indicate the maxillary molar extraction (CE and CAPE). (**c**) The amount of feed (in grams). (**d**) The amount of drinking water (in grams). (**e**) Serum albumin levels (g/dL). (**f**) Total cholesterol (T-CHO) levels (mg/dL) at the third month of rearing. CC, control diet with upper molars intact group; CE, control diet with upper molars extracted group; and CAPE, capsaicin diet with upper molars extracted group. One-way ANOVA, Tukey’s multiple comparisons test * *p* < 0.05, ** *p* < 0.01, *** *p* < 0.001, # *p* < 0.05, ## *p* < 0.01, ### *p* < 0.001. * corresponds to CC vs. CE and CC vs. CAPE; # corresponds to CE vs. CAPE. Results are presented as the mean ± SE. SE, standard error; ANOVA, analysis of variance.

We examined the body weight of aged C57BL/6N mice (control, CC), aged C57BL/6N mice (CE) that underwent teeth extraction and were kept on a control diet, and aged C57 BL/6N mice (CAPE) that underwent teeth extraction and consumed capsaicin food to investigate the effects of long-term feeding of capsaicin on the memory and learning functions of mice with impaired masticatory function (Figure 1a,b). CE and CAPE mice with extracted molars showed no significant changes in body weight from rearing to three months following extraction, while the CAPE group demonstrated slight weight loss immediately after extraction, followed by a steady increase in body weight (Figure 1b).

Feed and water intakes were also evaluated at 3 months following tooth extraction of rearing (Figure 1c,d). The daily feed intake 3 months after tooth extraction was higher in the CC (2.16 ± 0.14 g), CE (1.78 ± 0.2 g), and CAPE (3.34 ± 0.41 g) groups, with the CAPE group having a higher daily intake than that of the CC and CE group (CC vs. CAPE, *p* = 0.03, CE vs. CAPE, *p* < 0.01; (F (2, 9) = 8.712; Figure 1c). The volume of drinking water was also higher in the CAPE group. The difference between the CE and CAPE groups was significant (CC: 3.32 ± 0.36 g, CE: 2.54 ± 0.13 g, CAPE: 4.72 ± 0.53 g; CC vs. CE: *p* = 0.3, CC vs. CAPE: *p* < 0.05, CE vs. CAPE: *p* < 0.01; F (2, 9) = 8.617; Figure 1d).

Biochemical tests of the serum components were subsequently performed 3 months after tooth extraction: Albumin (ALB) (g/dL) did not differ between the groups (CC vs. CE: *p* = 0.88, CC vs. CAPE: *p* = 0.6, CE vs. CAPE: *p* = 0.88, F (2, 9) = 0.4848; Figure 1e); the T-CHO amount was also similar (CC vs. CE: *p* = 0.98, CC vs. CAPE: *p* = 0.45, CE vs. CAPE: *p* = 0.54; F (2, 9) = 0.9451; Figure 1f). Tooth extraction and capsaicin rearing had no effect on the nutritional status during the third month of rearing.

### 3.2. Effects of Missing Maxillary Molars and Capsaicin Feeding on the Memory and Behavior of Mice

#### 3.2.1. Twenty-Four-Hour Locomotor Activity

**Figure 2:**
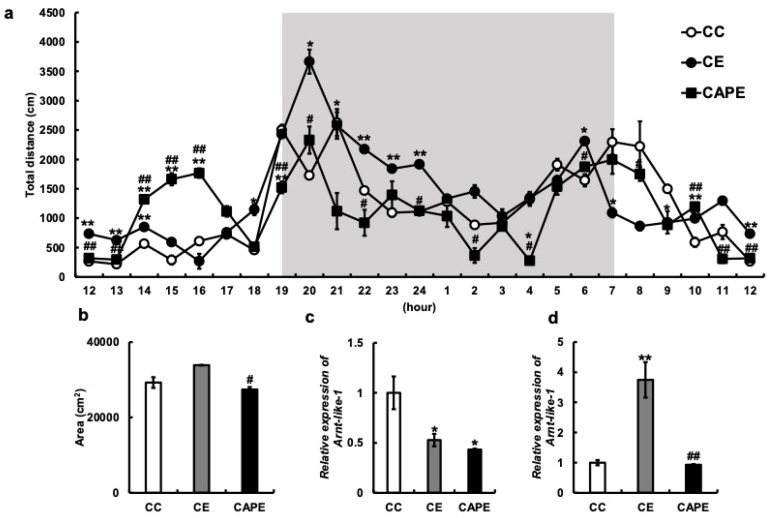
Effects of missing maxillary molars and capsaicin feeding on the memory and behavior in mice. The vertical axis represents the average total distance (cm) per hour. The horizontal axis indicates the time, with C = 1. Gray areas have lights off from 7:00 p.m. to 7:00 a.m. (**a**) Behavioral distance of each mouse for 24 h. (**b**) The behavioral distance for each mouse is presented as the area. (**c**) Expression of Aryl hydrocarbon receptor nuclear translocator-like protein 1 (Arnt-like 1) (*Bmal1)* mRNA in the hypothalamus. (**d**) *Bmal1* mRNA expression in the hippocampus; all the data are presented at 3 months. CC, control diet with upper molars intact group; CE, control diet with upper molars extracted group; CAPE, capsaicin diet with upper molars extracted group. One-way ANOVA, Tukey’s multiple comparisons test * *p* < 0.05, ** *p* < 0.01, *** *p* < 0.001, # *p* < 0.05, ## *p* < 0.01, ### *p* < 0.001. * corresponds to CC vs. CE and CC vs. CAPE; # corresponds to CE vs. CAPE. Results are presented as the mean ± SE. SE, standard error; ANOVA, analysis of variance; and mRNA, messenger ribonucleic acid.

Locomotion in the rearing cage was measured using an infrared sensor, and the total distance traveled per hour was calculated (Figure 2a). The CE group demonstrated a slight but not significant increase in the total distance traveled compared to the CC group (CC vs. CE: *p* = 0.08, Figure 2b), especially after 8:00 p.m., indicating increased activity. In contrast, the CAPE group demonstrated a decrease in nighttime activity compared to the CE group. An increase in the daytime (2:00–4:00 p.m.) activity was also observed in the CAPE group; however, the total distance traveled in 24 h was significantly lower in the CAPE group than in the CE group (CE vs. CAPE: *p* = 0.01, Figure 2b). These results suggested that the masticatory dysfunction increases nocturnal activity; however, it was suppressed by capsaicin administration.

To further investigate the increase in nocturnal activity owing to reduced masticatory function and its suppression in the CAPE group, we examined the changes in gene expression in the hypothalamus and hippocampus. A core clock gene aryl hydrocarbon receptor nuclear translocator-like protein 1 (Arnt-like 1) (*Bmal1)* is reportedly associated with circadian locomotor behavior [33]. Therefore, we examined *Bmal1* messenger (m)RNA expression in the brain. The results demonstrated that *Bmal1* mRNA expression in the hypothalamus was significantly decreased in both the CE and CAPE groups compared to the CC group (CC vs. CE: *p* = 0.02, CC vs. CAPE: *p* = 0.01, CE vs. CAPE: *p* = 0.83; F (2, 4) = 16.95, Figure 2c), while *Bmal1* mRNA was markedly increased in the hippocampus in the CE group compared to the CC group (CC vs. CE: *p* < 0.01, CC vs. CAPE: *p* = 0.98, CE vs. CAPE: *p* < 0.01; F (2, 5) = 25.99, Figure 2d).

#### 3.2.2. Memory and Behavior

**Figure 3:**
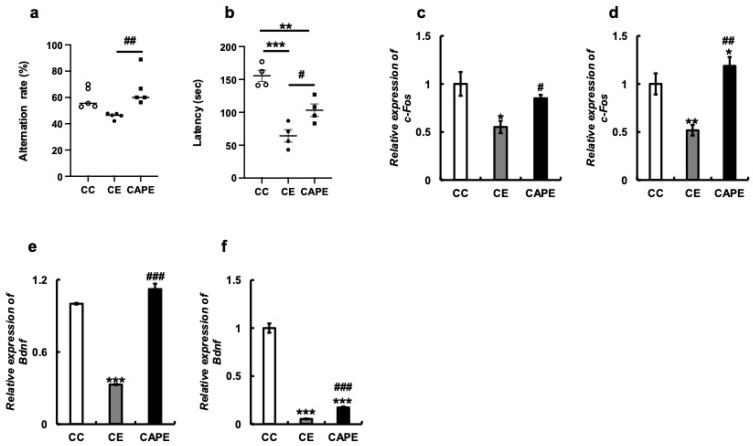
Effect of missing molars rearing on mice behavior. (**a**) Alternation rate in the Y maze test (%). (**b**) Latency in the rotarod (seconds). (**c**) mRNA expression of *c-Fos* in the hypothalamus. (**d**) mRNA expression of *c-Fos* in the hippocampus. (**e**) mRNA expression of *Bdnf* in the hypothalamus. (**f**) mRNA expression of *Bdnf* in the hippocampus. All the data are presented at three months. CC, control diet with upper molars intact group; CE, control diet with upper molars extracted group; CAPE, capsaicin diet with upper molars extracted group. In this study, the comparison between the groups (CC, CE, and CAPE) was performed using statistical analyses, including Tukey’s post hoc test for multiple comparisons. The significance levels between the groups are indicated (* *p* < 0.05, ** *p* < 0.01, *** *p* < 0.001, # *p* < 0.05, ## *p* < 0.01, ### *p* < 0.001). The results are presented as mean ± standard error.

The loss of molars reportedly results in cognitive decline in mice [3,34,35]. The Y-maze test was used to assess spatial working memory in mice. The CE group demonstrated a lower alternation rate compared to the CC group (*p* = 0.08), but the difference was not statistically significant. Conversely, the CAPE group exhibited a significantly greater recovery in spatial working memory compared to the CE group (*p* < 0.01; F (2, 12) = 6.647; Figure 3a). The effect of tooth extraction on motor learning function was examined using the rotarod test. Latency in the rotarod test was significantly shorter in the CE group than in the CC group (CC vs. CE: *p* < 0.001, Figure 3b). In the CAPE group, the latency was slightly longer (CE vs. CAPE: *p* < 0.05, Figure 3b), impairing the balance and motor coordination in the CE group by rotarod testing, which was restored by capsaicin administration. (F (2, 9) = 24.79).

Masticatory function impairment in mice is associated with impaired cognitive function [2,3]. We confirmed *c-Fos* mRNA expression in the hypothalamus and hippocampus. In the hypothalamus, *c-Fos* was significantly decreased in the CE group compared to the CC group (*p* < 0.05) and significantly recovered in the CAPE group (*p* = 0.02; F (2, 6) = 6.098; Figure 3c). Similar results were obtained for the hippocampus (CC vs. CE: *p* < 0.01, CC vs. CAPE: *p* = 0.025, CE vs. CAPE: *p* < 0.01; F (2, 8) = 14.99, Figure 3d). 

In the hypothalamus, the mRNA expression of brain-derived neurotrophic factor (Bdnf), which is required for the establishment and maintenance of memory and learning, was significantly lower in the CE group than in the CC group (*p* < 0.001) and recovered in the CAPE group (*p* < 0.001, (F (2, 4) = 123.5, Figure 3e). Similarly, Bdnf expression in the hippocampus was significantly restored in the CAPE group (*p* < 0.001) compared to that in the CC and CE groups (F (2, 7) = 230.5, Figure 3f).

#### 3.2.3. Microglia- and Neuron-Associated mRNA and Protein Expression in the Hypothalamus and Hippocampus

**Figure 4:**
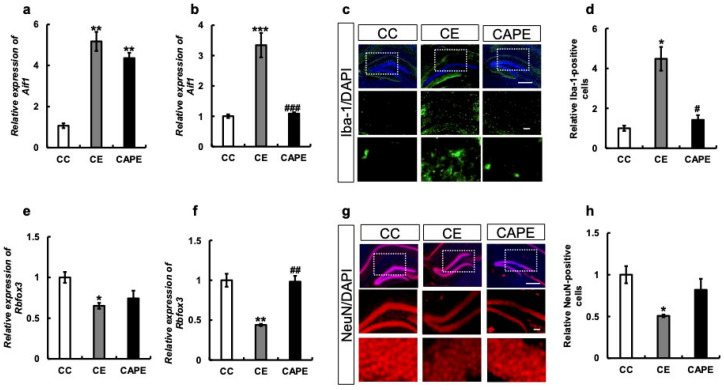
Microglia- and Neuron-Associated mRNA and Protein Expression in the Hypothalamus and Hippocampus. (**a**) *Aif1* mRNA expression in the hypothalamus. (**b**) *Aif1* mRNA expression in the hippocampus. (**c**) Iba-1 immunostaining in the hippocampus. Each group is shown vertically: Overall view of the hippocampus in each group. Enlarged view of the dotted line area in each group. Enlarged view of characteristic parts in each group. (**d**) Iba-1 positive cells in the hippocampus. The y-axis of the graph represents the ratio of Iba-1 expression normalized to the DAPI signal, with the control set as 1. (**e**) *Rbfox3* (NeuN) mRNA expression in the hypothalamus. (**f**) *Rbfox3* (NeuN) mRNA expression in the hippocampus. (**g**) NeuN immunostaining in the hippocampus. Each group is shown vertically: Overall view of the hippocampus in each group. Enlarged view of the dotted line area in each group. Enlarged view of characteristic parts in each group. (**h**) NeuN-positive cells in the hippocampus. The y-axis of the graph represents the ratio of the NeuN expression normalized to the DAPI signal, with the control set as 1. All the data are presented at three months. CC, control diet with upper molars intact group; CE, control diet with upper molars extracted group; CAPE, capsaicin diet with upper molars extracted group. Scale bar, 100 μm. One-way ANOVA, Tukey’s multiple comparisons test * *p* < 0.05, ** *p* < 0.01, *** *p* < 0.001, # *p* < 0.05, ## *p* < 0.01, ### *p* < 0.001. * corresponds to CC vs. CE and CC vs. CAPE; # corresponds to CE vs. CAPE. Results are presented as the mean ± SE. SE, standard error; ANOVA, analysis of variance; and mRNA, messenger ribonucleic acid.

We examined the expression of microglial marker allograft inflammatory factor 1 (AIF1) (ionized calcium-binding adapter molecule 1, Iba-1) at the transcriptional level in the hypothalamus and hippocampus. We observed a significant increase in Aif1 mRNA expression in the hypothalamus of mice in the CE group compared to the CC group (CC vs. CE: *p* < 0.01; F (2, 4) = 63.04; Figure 4a). However, there was a slight suppression of Aif1 mRNA expression in the CAPE group, although the difference was not statistically significant (CE vs. CAPE: *p* = 0.1676; Figure 4a). In the hippocampus, Aif1 expression was significantly increased in the CE group and suppressed in the CAPE group compared to the CC group (CC vs. CE: *p* < 0.001, CC vs. CAPE: 0.9247, CE vs. CAPE: *p* < 0.001; F (2, 5) = 55.68; Figure 4b). In addition, immunostaining of the hippocampus demonstrated more Iba-1-positive cells in the CE group and similar levels in the CC and CAPE groups (CC vs. CE: *p* = 0.02, CC vs. CAPE: *p* = 0.26, CE vs. CAPE: *p* = 0.03, (F (2, 4) = 16.36), Figure 4c,d).

Since inflammation is known to result in neuronal damage [36], neuronal nuclei (NeuN) were examined. In the hypothalamus, the mRNA expression of RBFOX3 (NeuN), a marker of neurons, was lower in the CE group compared to the CC group (CC vs. CE: *p* < 0.05, Figure 4e). Additionally, there was a slight trend of recovery in RBFOX3 mRNA expression in the CAPE group, although the difference was not statistically significant (CE vs. CAPE: *p* = 0.62; F (2, 5) = 9.221, Figure 4e). In the hippocampus, the recovery of the CAPE group was similar to that of the CC group (CC vs. CE: *p* < 0.01, CE vs. CAPE: *p* < 0.01; F (2, 6) = 24.98), Figure 4f). Similar results were obtained for protein expression (Figure 4g,h). Tooth extraction suggested microglial activity and nerve damage.

#### 3.2.4. Astrocyte-Related mRNA and Protein Expression in the Hypothalamus and Hippocampus

**Figure 5:**
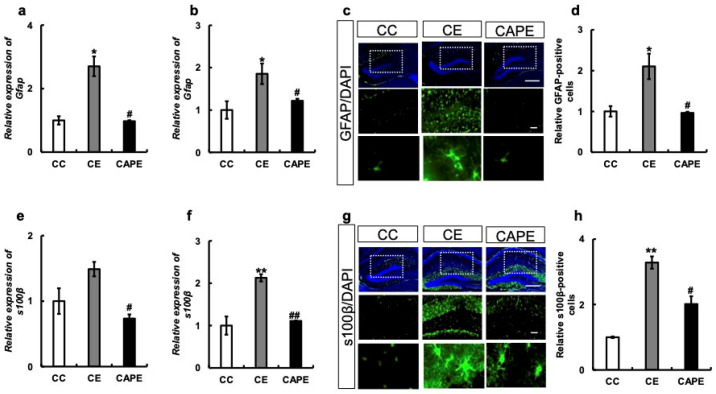
Astrocyte-related mRNA and protein expression in the hypothalamus and hippocampus. (**a**) *Gfap* mRNA expression in the hypothalamus. (**b**) *Gfap* mRNA expression in the hippocampus. (**c**) *Gfap* immunostaining in the hippocampus. Each group is shown vertically: Overall view of the hippocampus in each group. Enlarged view of the dotted line area in each group. Enlarged view of characteristic parts in each group. (**d**) GFAP-positive cells in the hippocampus. The y-axis of the graph represents the ratio of the GFAP expression normalized to the DAPI signal, with the control set as 1. (**e**) *s100β* mRNA expression in the hypothalamus. (**f**) *s100β* mRNA expression in the hippocampus. (**g**) s100β immunostaining in the hippocampus. Each group is shown vertically: Overall view of the hippocampus in each group. Enlarged view of the dotted line area in each group. Enlarged view of characteristic parts in each group. (**h**) s100β positive cells in the hippocampus. The y-axis of the graph represents the ratio of the s100β expression normalized to the DAPI signal, with the control set as 1. All the data are presented at three months. CC, control diet with upper molars intact group; CE, control diet with upper molars extracted group; CAPE, capsaicin diet with upper molars extracted group. Scale bar, 100 μm. One-way ANOVA, Tukey’s multiple comparisons test * *p* < 0.05, ** *p* < 0.01, *** *p* < 0.001, # *p* < 0.05, ## *p* < 0.01, ### *p* < 0.001. * corresponds to CC vs. CE and CC vs. CAPE; # corresponds to CE vs. CAPE. Results are presented as the mean ± SE. SE, standard error; ANOVA, analysis of variance; and mRNA, messenger ribonucleic acid.

We examined the expression of GFAP, a marker of astrocyte senescence, at the mRNA level in the hypothalamus and hippocampus. The results demonstrated that *Gfap* mRNA expression was significantly increased in the hippocampus of mice in the CE group compared to mice in the CC group (CC vs. CE: *p* = 0.019; Figure 5a), which was suppressed to the same extent as in the CAPE and CC groups (CE vs. CAPE: *p* = 0.02; F (2, 4) = 16.49, Figure 5a). Similar results were obtained in the hippocampus (F (2, 7) = 5.142, Figure 5b). Furthermore, in hippocampal immunostaining, a higher number of GFAP-positive cells was observed in the CE group compared to both the CC group and the CAPE group (F (5, 18) = 11.29), Figure 5c,d).

The mRNA expression of s100β, a calcium-binding protein specifically expressed in astrocytes, showed a tendency to increase in the CE group compared to the CC group in the hypothalamus, but the difference was not statistically significant (CC vs. CE: *p* = 0.09, Figure 5e). However, in the CAPE group, the mRNA expression of s100β was suppressed (CE vs. CAPE: *p* = 0.03; F (2, 6) = 6.845, Figure 5e). Similar results were obtained in the hippocampus (F (2, 4) = 28.85, Figure 5f) and for protein expression (F (2, 6) = 23.70), Figure 5g,h).

#### 3.2.5. AMPA-Related and Trpv1 mRNA and Protein Expression in the Hypothalamus and Hippocampus

**Figure 6:**
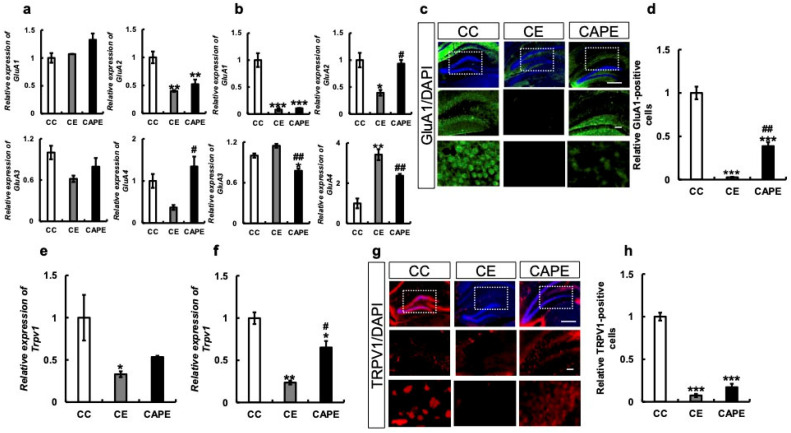
AMPA-related and TRPV1 mRNA expression and protein expression in the hypothalamus and hippocampus. (**a**) *GluA1-GluA4* mRNA expression in the hypothalamus. (**b**) *GluA1-GluA4* mRNA expression in the hippocampus. (**c**) GluA1 immunostaining in the hippocampus. Each group is shown vertically: Overall view of the hippocampus in each group. Enlarged view of the dotted line area in each group. Enlarged view of characteristic parts in each group. (**d**) GluA1 positive cells in the hippocampus. The y-axis of the graph represents the ratio of the AMPA expression normalized to the DAPI signal, with the control set as 1. (**e**) *Trpv1* mRNA expression in the hypothalamus. (**f**) *Trpv1* mRNA expression in the hippocampus. (**g**) TRPV1 immunostaining in the hippocampus. Each group is shown vertically: Overall view of the hippocampus in each group. Enlarged view of the dotted line area in each group. Enlarged view of characteristic parts in each group. (**h**) TRPV1 positive cells in the hippocampus. The y-axis of the graph represents the ratio of the TRPV1 expression normalized to the DAPI signal, with the control set as 1. All the data are presented at 3 months of rearing. CC, control diet with upper molars intact group; CE, control diet with upper molars extracted group; CAPE, capsaicin diet with upper molars extracted group. Scale bar, 100 μm. One-way ANOVA, Tukey’s post hoc test: * *p* < 0.05, ** *p* < 0.01, *** *p* < 0.001, # *p* < 0.05, ## *p* < 0.01, ### *p* < 0.001. * corresponds to CC vs. CE and CC vs. CAPE; # corresponds to CE vs. CAPE. In this study, the comparison between the groups (CC, CE, and CAPE) was performed using statistical analyses, including Tukey’s post hoc test for multiple comparisons. One-way ANOVA, Tukey’s multiple comparisons test * *p* < 0.05, ** *p* < 0.01, *** *p* < 0.001, # *p* < 0.05, ## *p* < 0.01, ### *p* < 0.001. * corresponds to CC vs. CE and CC vs. CAPE; # corresponds to CE vs. CAPE. Results are presented as the mean ± SE. SE, standard error; ANOVA, analysis of variance; mRNA, messenger ribonucleic acid; AMPA, α-amino-3-hydroxy-5-methyl-4-isoxazole propionic acid-type glutamate receptor; TRPV1, transient receptor potential vanilloid 1; and GluA, glutamate ionotropic receptor AMPA-type unit.

We examined the mRNA expression of the AMPA receptor subunit, a molecule responsible for brain function in the hypothalamus and hippocampus. The results demonstrated that GluA1 was unchanged in each group (CC vs. CE: *p* = 0.84, CC vs. CAPE: *p* = 0.09, CE vs. CAPE: *p* = 0.14; F (2, 5) = 4.328, Figure 6a), while GluA2 was significantly decreased in the CE group and recovered in the CAPE group (CC vs. CE: *p* < 0.01, CC vs. CAPE: *p* = 0.01, CE vs. CAPE: *p* = 0.54, F (2, 7) = 14.61, Figure 6a) in the hypothalamus; GluA3 was decreased in the CE group and recovered in the CAPE group, but not significantly. (CC vs. CE: *p* = 0.1, CC vs. CAPE: *p* = 0.39, CE vs. CAPE: *p* = 0.48, F (2, 7) = 2.933, Figure 6a). GluA4 levels decreased in the CE group but not significantly, and fully recovered in the CAPE group (CC vs. CE: *p* = 0.2, CC vs. CAPE: *p* = 0.52, CE vs. CAPE: *p* = 0.02; F (2, 6) = 6.652, Figure 6a).

In the hippocampus, GluA1 expression was significantly decreased in the CE and CAPE groups compared to that in the CC group (CC vs. CE: *p* < 0.001, CC vs. CAPE: *p* < 0.001, CE vs. CAPE: *p* = 0.96; F (2, 6) = 48.85, Figure 6b). On the other hand, the mRNA expression of GluA2 decreased in the CE group and recovered in the CAPE group (CC vs. CE: *p* = 0.025, CC vs. CAPE: *p* = 0.89, CE vs. CAPE: *p* = 0.03 (F (2, 5) = 8.708, Figure 6b). 

GluA3 mRNA expression increased in the CE group but not significantly and decreased in the CAPE group (CC vs. CE: *p* = 0.058, CC vs. CAPE: *p* = 0.02, CE vs. CAPE: *p* < 0.01; F (2, 4) = 36.83, Figure 6b). GluA4 was markedly increased in the CE group and decreased in the CAPE group (CC vs. CE: *p* < 0.01, CC vs. CAPE: *p* = 0.96, CE vs. CAPE: *p* < 0.01; F (2, 7) = 15.53), Figure 6b). Immunostaining for GluA1 in the hippocampus demonstrated a decrease in the number of positive cells in the CE group and recovery of positive cells in the CAPE group (CC vs. CE: *p* < 0.001, CC vs. CAPE: *p* < 0.001, CE vs. CAPE: *p* < 0.01; Figure 6c,d).

The mRNA expression of TRPV1, a receptor for capsaicin, decreased in the hypothalamus of the CE group compared to the CC group (CC vs. CE: *p* = 0.04), and in the CAPE group, there was a slight recovery, although the difference was not statistically significant (CE vs. CAPE: *p* = 0.54; F (2, 4) = 7.105, Figure 6e). In the hippocampus, the CE group showed a decrease compared to the CC group, and the CAPE group demonstrated recovery (F (2, 5) = 27.86, Figure 6f). Immunostaining of the hippocampus showed a significant decrease in TRPV1-positive cells in the CE group and a slight recovery in the CAPE group, but the difference was not statistically significant (CC vs. CE: *p* < 0.001, CC vs. CAPE: *p* < 0.001, CE vs. CAPE: *p* = 0.37; F (5, 13) = 77.36, Figure 6g,h).

#### 3.2.6. Expression of Gfap and Trpv1 mRNA in Cultured Astrocytes

Cultured mouse astrocytes were classified into three groups: control (C), aged astrocytes (H), and aged astrocytes with 25 μM capsaicin (H + C25).

**Figure 7:**
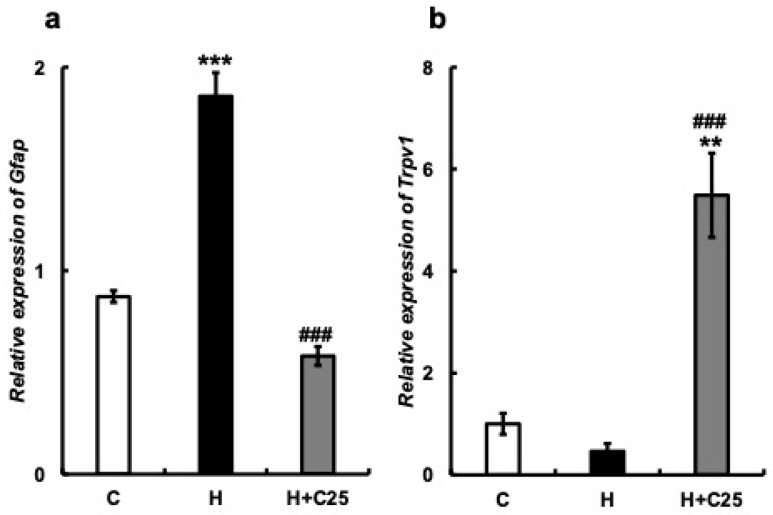
The addition of capsaicin to cultured mouse astrocytes suppresses GFAP expression. (**a**) *Gfap* mRNA expression in the cultured mouse astrocytes. 75 μM H_2_O_2_ induced GFAP expression, and an additional 25 μM of capsaicin suppressed GFAP expression. (**b**) *Trpv1* mRNA expression in the cultured mouse astrocytes. 75 μM H_2_O_2_ reduced *Trpv1* expression, and an additional 25 μM of capsaicin increased *Trpv1* expression. C, control mouse astrocytes; H, hydrogen peroxide-induced senescent astrocytes; and H + C25, senescent astrocytes with hydrogen peroxide and additional 25 µM capsaicin. One-way ANOVA, Tukey’s multiple comparisons test * *p* < 0.05, ** *p* < 0.01, *** *p* < 0.001, # *p* < 0.05, ## *p* < 0.01, ### *p* < 0.001. * corresponds to C vs. H and H vs. H + C25; # corresponds to H vs. H + C25. Results are presented as the mean ± SE. SE, standard error; ANOVA, analysis of variance; and mRNA, messenger ribonucleic acid.

Astrocytes induce cellular senescence using H_2_O_2_ [32]. *Gfap* mRNA expression was significantly increased in the H group. The addition of 25 μM of capsaicin suppressed *Gfap* mRNA expression (C vs. H: *p* < 0.001, C vs. H + C25: *p* = 0.65, H vs. H + C25: *p* < 0.001; F (2, 6) = 106.7; Figure 7a). The *Trpv1* mRNA levels were reduced in the H group and increased in the H + C25 group (C vs. H: *p* = 0.74, C vs. H + C25: *p <* 0.01, H vs. H + C25: *p* < 0.001; F (2, 6) = 30.46; Figure 7b).

## 4. Discussion

We found that molar extraction altered locomotor activity and gene expression related to cognitive function in the hypothalamus and hippocampus of aged mice with impaired masticatory function, which were ameliorated by the long-term administration of capsaicin. In addition, the expression of TRPV1, a receptor for capsaicin, was downregulated in mice with missing molars. However, long-term capsaicin administration resulted in the normalization of nocturnal activity and dementia-related gene expression. In addition, TRPV1 expression in the hypothalamus and hippocampus was restored in the capsaicin-treated mice. Since TRPV1 signaling in the brain is reportedly important for the maintenance of cognitive function in mouse models [26], improvement in brain function by capsaicin may involve the enhancement of TRPV1 signaling. Our study demonstrated that TRPV1 prevented cognitive decline owing to loss of masticatory function and suggested that TRPV1 may be an effective molecular target and its agonist, capsaicin, may be applied to maintain brain function in patients who are bedridden or have dementia and have difficulty receiving prosthetic treatment.

An abnormal increase in the number of astrocytes in the brain is known as astrogliosis [14]. Astrogliosis exists in various forms with varying intensities, such as increased GFAP expression and cell hypertrophy. Recently, we demonstrated that astrogliosis is caused by loss of masticatory function [3]. In addition, astrogliosis is also associated with cognitive dysfunction and behavioral abnormalities [37]. 

In this study, we extracted maxillary molars from mice to simulate impaired masticatory function. The healing process after tooth extraction typically takes 1–2 weeks [35]. In this experiment, it is unlikely that bacterial infection resulting from maxillary molar extraction in mice led to neuroinflammation. Moreover, no significant issues were observed in the behavioral experiments or subsequent studies. Previous reports have demonstrated temporary weight loss immediately after tooth extraction; however, no significant changes in body weight were observed thereafter [3,35].

The literature suggests that when capsaicin or chili peppers are provided with meals, their pungency increases satiety [38,39]. However, in this study, CAPE mice consumed more feed than the CC or CE mice (Figure 1c). Although the capsaicin content may have decreased the feed intake, the CAPE group showed no effect on the food intake. This suggested that mice do not avoid capsaicin-containing feed. This is consistent with the results of previous studies [40,41]. An 8-month subchronic feeding study with capsaicin did not observe any toxicity. The CAPE group originally consisted of mice that weighed less than those of the other groups (Figure 1b). Their body weight was not reduced owing to diet or tooth extraction, and they eventually gained weight since they were still able to consume feed after tooth extraction. Therefore, the safety of capsaicin is ensured when consumed at concentrations of 0.003–0.03% of the diet [25]. 

In addition, Sprague Dawley rats treated with a capsaicin water suspension regulated the expression of the circadian clock gene Per2 [42]. Capsaicin regulates the rhythmic expression of the circadian clock gene Bmal1 in in vitro experiments using human hepatocellular carcinoma cells [43]. In our study, the CAPE-treated group alleviated the nocturnal and overall activity levels increased by tooth extraction to the same extent as the CC group (Figure 2a,b). In the hippocampus, the CAPE group suppressed the increased expression of Bmal1 in the CE group (Figure 2d). However, the CAPE group failed to alter its expression in the hypothalamus, which was reduced in the CE group (Figure 2c). The suprachiasmatic nucleus in the hypothalamus plays an important role in the regulation of circadian rhythm. However, in our study, we measured the expression of Bmal1 mRNA across the entire hypothalamus. As a result, we were unable to detect any differences in gene expression specifically within the suprachiasmatic nucleus. In contrast, Bmal1 expression in the hippocampus is involved in memory retrieval [44,45]. Therefore, altered Bmal1 gene expression resulting from molar loss may have been associated with cognitive decline in this study.

S100β, which is a protein expressed and released from the astrocytes, is involved in neuroinflammation and degeneration and is often involved in the central nervous system [46]. Increased S100β release may be involved in the memory deficits seen early in AD [47], and S100β expression in the hippocampus increases with molar loss [9]. Among the various signaling molecules secreted by astrocytes and released during reactive gliosis occurring in AD, astrocyte-derived S100β protein plays an important role in neuroinflammation, one of the hallmarks of this disease [46,47].

In this study, the CE group demonstrated a decrease in NeuN and an increase in IBA1 expression, suggesting microglial activation and neuroinflammation (Figure 4). However, these effects were reversed by capsaicin administration.

The AMPA-type glutamate is responsible for rapid neurotransmission in the central nervous system [48]. Glutamate and AMPA receptor-mediated signal transduction, termed synaptic plasticity, is considered a fundamental process in memory and learning [49,50]. Recent studies have demonstrated that the changes in the number of AMPA receptors in the dendrites are molecular processes of synaptic plasticity [50]. For example, long-term depression, a form of synaptic plasticity, is the long-term decrease in the efficiency of information transmission at the synapse caused by a decrease in the number of AMPA receptors in the dendrites [50]. The AMPA receptor has four subunits, GluA1, GluA2, GluA3, and GluA4, which form homo- or heterotetramers. GluA2 is an important subunit determining AMPA receptor function. In this study, a marked decrease in the GluA2 mRNA levels was observed in mice with missing molars, indicating that the AMPA receptor signaling-mediated responses were reduced in these mice (Figure 6a,b). However, its expression was restored by capsaicin administration, suggesting that capsaicin could improve various AMPA receptor-mediated brain functions. A decrease in coordinated motor function was observed in mice with missing molars. In contrast, capsaicin-treated mice demonstrated an improvement in motor function. Additionally, a decrease in GluA4 mRNA expression in the hypothalamus was observed in molar-depleted mice, which was restored by capsaicin treatment. Since impaired motor function has been observed in GluA4-deficient mice [51,52], decreased GluA4 expression may be responsible for impaired motor coordination. In addition, motor coordination improvement by capsaicin administration could restore GluA4 expression.

One possible cause of brain dysfunction attributed to decreased masticatory function is the weakening of nociceptor stimulation in the periodontal ligament. Thus, the weakening of signals projected via the trigeminal nerve to the cerebral cortex and hippocampus may result in changes in brain function. However, since TRPV1 is widely distributed in the gastrointestinal mucosa, including the oral mucosa, capsaicin ingested through the oral cavity activates its receptors on the mucosal surface. The impulse is then transmitted through the spinal cord and medulla oblongata to the thalamus, hypothalamus, and midbrain and then projected to the hippocampus and cerebral cortex, where it is reflected in gene expression and brain functions. Therefore, the effect of capsaicin observed in this study may be attributed to capsaicin stimulation compensating for the reduced stimulation of the periodontal ligament. However, in in vitro experiments, low concentrations of H_2_O_2_-induced senescence of cultured astrocytes, that is, Gfap mRNA expression, were inhibited by the addition of capsaicin. Moreover, Trpv1 mRNA levels decreased with aging and increased with capsaicin addition (Figure 7). This may play a role in elucidating the mechanism by which molar loss in aged mice showed a cognitive decline in vivo, which was improved in capsaicin-fed mice. Capsaicin also possibly migrates to the brain and expresses TRPV1 via the blood-brain barrier [53]. The effects of capsaicin intake on TRPV1 expression levels have already been reviewed [54].

**Figure 8:**
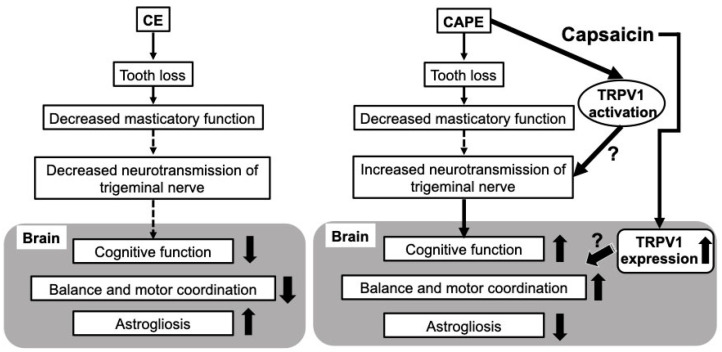
Capsaicin may restore lost function via TRPV1. Decreased mastication leads to cognitive decline and astrogliosis. Capsaicin may improve its function via TRPV1. CE, control diet with upper molars extracted group; CAPE, capsaicin diet with upper molars extracted group; and TRPV1, transient receptor potential vanilloid 1.

Most clinical treatment goals are the improvement in the masticatory function through prophylaxis, oral care, or prosthetic treatment to prevent cognitive decline owing to loss of molar teeth. However, since the number of patients with dementia and bedridden patients increases each year, prosthetic treatment will become more challenging in the future. This study suggests that capsaicin, a TRPV1 agonist, may be effective not only in promoting the swallowing reflex and as a potential AD treatment [26] but also in patients with cognitive decline and difficulty with oral prosthetic therapy. Thus, capsaicin can inhibit the decline in brain function, including cognitive function, in patients with various diseases that make prosthetic treatment challenging (Figure 8). 

## Figures and Tables

**Table 1:** Sequences of primers used for real-time polymerase chain reaction.

Name	Forward	Reverse
Mouse *Arnt-like 1* (Bmal1)	TGCCACCAATCCATACACAG	TTCCCTCGGTCACATCCTAC
Mouse Fos (*c-Fos*)	GGGGACAGCCTTTCCTACTA	CTGTCACCGTGGGGATAAAG
Mouse *Bdnf*	TGCAGGGGCATAGACAAAAGG	CTTATGAATCGCCAGCCAATTCTC
Mouse *Aif1* (Iba-1)	CTTTTGGACTGCTGAAGGC	GTTTCTCCAGCATTCGCTTC
Mouse *Rbfox3* (NeuN)	CACCACTCTCTTGTCCGTTTGC	GGCTGAGCATATCTGTAAGCTGC
Mouse *Gfap*	TCCTGGAACAGCAAAACAAG	CAGCCTCAGGTTGGTTTCAT
Mouse *s100β*	CCCTCATTGATGTCTTCCACC	TCTCCATCACTTTGTCCACC
Mouse *GluA1*	GGACAACTCAAGCGTCCAGA	GTCGGTAGGAATAGCCCACG
Mouse *GluA2*	GCGTGGAAATAGAAAGGGCC	ACTCCAGTACCCAATCTTCCG
Mouse *GluA3*	ACCATCAGCATAGGTGGACTT	ACGTGGTAGTTCAAATGGAAGG
Mouse *GluA4*	GGCCAGGGAATTGACATGGA	CCTTTCGAGGTCCTGTGCTT
Mouse *Trpv1*	CATCTTCACCACGGCTGCTTAC	CAGACAGGATCTCTCCAGTGAC
Mouse *Gapdh*	AACCTGCCAAGTATGATGA	GGAGTTGCTGTTGAAGTC

Abbreviations: Bmal1, aryl hydrocarbon receptor nuclear translocator-like protein 1 (Arnt-like 1); *Rbfox3*, RNA binding protein fox-1 homolog 3; NeuN, neuronal nuclei; *Bdnf*, brain-derived neurotrophic factor; *Gfap*, glial fibrillary acidic protein; *s100β*, S100 calcium-binding protein beta; *GluA1*, glutamate ionotropic receptor AMPA-type subunit 1; *GluA2*, glutamate ionotropic receptor AMPA-type subunit 2; *GluA3*, glutamate ionotropic receptor AMPA-type subunit 3; *GluA4*, glutamate ionotropic receptor AMPA-type subunit 4; and *Trpv1*, transient receptor potential vanilloid-1.

## Data Availability

The datasets used and/or analyzed during this study are available from the corresponding authors upon reasonable request.
